# CSE‐Derived Hydrogen Sulfide Safeguards Vascular Iron Homeostasis by Coupling Ferritin Buffering to Vasoprotection

**DOI:** 10.1096/fj.202502763RR

**Published:** 2025-10-21

**Authors:** Hassan Mustafa Arif, Ming Fu, Richa Verma, Rui Wang

**Affiliations:** ^1^ Department of Biology York University Toronto Ontario Canada; ^2^ College of Basic Medicine Shandong Second Medical University Weifang China

**Keywords:** hydrogen sulfide, hypertension, iron overload, oxidative stress, vascular dysfunction

## Abstract

Iron overload drives oxidative stress and vascular injury, yet the role of hydrogen sulfide (H_2_S) in systemic and vascular iron regulation remains unclear. We investigated how cystathionine γ‐lyase (CSE)‐derived H_2_S influences iron handling and vascular responses following acute iron overload. Wild‐type (WT) and CSE‐knockout (CSE‐KO) mice received PBS, low‐concentration (1×) or high‐concentration (3×) iron dextran intravenously. CSE‐KO mice exhibited elevated serum iron and transferrin saturation but impaired ferritin upregulation compared to WT. KO aortas showed greater iron deposition, elastin degradation, and inflammatory remodeling under high iron. Iron overload impaired phenylephrine‐induced vasocontraction and H_2_S‐mediated vasorelaxation, with CSE‐KO mice displaying the most severe vascular dysfunction and elevated blood pressure. WT mice compensated via CSE upregulation and increased H_2_S production. Our findings demonstrate that CSE/H_2_S deficiency disrupts ferritin‐mediated iron storage, exacerbates vascular iron accumulation, and worsens vasomotor dysfunction during iron overload. The CSE/H_2_S pathway may represent a therapeutic target for mitigating iron‐induced vascular damage.

## Graphical abstract

With iron overload, wild‐type (CSE^+^/^+^) mice upregulate their CSE and ferritin expression, limiting oxidative stress and maintaining vascular health. In contrast, CSE‐deficient (CSE^−^/^−^) mice fail to induce ferritin, leading to elevated oxidative stress, vascular remodeling, and hypertension. These findings highlight the protective role of the CSE/H_2_S pathway against iron‐induced vascular injury.

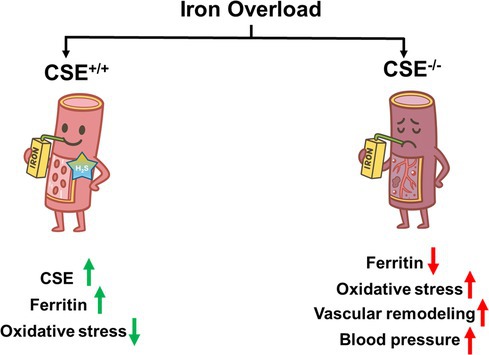

## 1 Introduction

Iron is an essential micronutrient required for critical biological processes such as oxygen transport, mitochondrial energy production, and maintenance of redox balance [1]. However, excessive iron can catalyze the generation of reactive oxygen species (ROS) via Fenton chemistry, leading to oxidative stress, cellular damage, and tissue injury. To prevent iron‐mediated toxicity, iron homeostasis is tightly regulated by systemic mechanisms—primarily the hepcidin–ferroportin axis—and by cellular sequestration of iron in ferritin [2]. Dysregulation of these pathways, as seen in hereditary hemochromatosis, transfusion‐related iron loading, or intravenous iron therapy in chronic kidney disease, has been linked to vascular dysfunction, arterial stiffness, and increased cardiovascular risk [1].

Hydrogen sulfide (H_2_S), a gaseous signaling molecule generated endogenously by cystathionine γ‐lyase (CSE), has emerged as a key regulator of vascular tone, redox balance, and anti‐inflammatory signaling [3]. Beyond its vasorelaxant properties, H_2_S is increasingly recognized as a modulator of iron metabolism, influencing iron regulatory proteins (IRPs), ferritin synthesis, and labile iron pools [4]. Our previous studies in vascular smooth muscle cells (VSMCs), along with recent findings by Zhu et al., demonstrated that H_2_S deficiency amplifies iron‐induced oxidative stress, limits ferritin upregulation, and increases cell death [4, 5]. Despite these insights, the in vivo role of the CSE/H_2_S axis in systemic iron regulation and vascular iron handling remains poorly understood.

Acute iron overload, such as that caused by intravenous iron administration or transfusion therapy, transiently exposes vascular tissues to elevated circulating iron before hepatic clearance occurs [1], potentially inducing oxidative damage. Whether H_2_S signaling counteracts this acute vascular iron stress in vivo, and how its deficiency impacts systemic iron homeostasis and vascular remodeling, is unknown. Addressing this gap may uncover novel therapeutic strategies to prevent iron‐induced vascular injury.

The aim of this study was to investigate how CSE/H_2_S deficiency influences systemic iron regulation, vascular iron accumulation, and functional vascular responses during acute iron overload in vivo. We hypothesized that the CSE/H_2_S pathway plays a critical role in modulating both systemic and vascular responses to iron loading.

## 2 Material and Methods

### 2.1 Animals and Protocol

All experimental procedures were approved by the Animal Care Committee at York University and followed the Canadian Council on Animal Care (CCAC) guidelines. Wild‐type (WT) mice on a C57BL/6J × 129SvEv background and in‐house‐bred homozygous cystathionine gamma‐lyase knockout (CSE‐KO) mice were used. Homozygous CSE‐KO mice were obtained by at least 10 generations of backcrossing heterozygous CSE‐KO mice with WT mice, as previously described [6]. All animals were housed in HVAC‐ventilated cages (4–5 mice per cage) maintained on a 12:12‐h light/dark cycle, at 21°C–23°C and 40%–60% humidity, with unrestricted access to autoclaved water and standard rodent chow. Cages were randomly assigned to experimental groups to reduce allocation bias.

Eight‐week‐old male WT and CSE‐KO mice were divided into three treatment groups (*n* = 6–8 per group):

(1) control, which received a phosphate buffered saline (PBS) injection (tail vein);
(2) low‐concentration iron (1× iron), which received a single 15 mg/kg tail‐vein injection of iron dextran;
(3) high‐concentration iron (3× iron), which received 15 mg/kg iron dextran administered three times, 2 h apart. The 3× iron dextran concentration approximates the pharmacological doses of intravenous iron used in the treatment of iron‐deficiency anemia [7].

All animals were euthanized 24 h after the initial injection for blood and tissue collection.

Mice exhibiting signs of tail‐vein occlusion, injection failure, or signs of distress were removed from the study. The sample size was chosen based on prior studies assessing H_2_S responses in atherosclerosis using our WT and CSE‐KO mice [8], which showed that a group size of 6–8 animals was sufficient to detect biologically significant differences with a power of 80% and *α* = 0.05.

All injections (PBS and iron dextran) were prepared using reagents from Sigma‐Aldrich (Oakville, Canada).

### 2.2 Serum Iron, TIBC, and Transferrin Saturation Measurement

Mice were euthanized 24 h after the initial injection, and blood was collected by performing cardiac puncture into plain Eppendorf tubes (without anticoagulant). The collected blood was allowed to clot for 30 min at room temperature, then centrifuged at 845 × g for 3 min to separate the serum, which was carefully transferred into fresh tubes for analysis. Serum iron and total iron‐binding capacity (TIBC) were determined using the TIBC and Serum Iron Assay Kit (Abcam, Concord, Canada; ab239715), following the manufacturer's protocol. Absorbance for all standards and samples was recorded at 570 nm using a Biotek Synergy HTX multimode plate reader (Agilent, Mississauga, ON, Canada). All measurements were performed in duplicate, and any sample showing visible hemolysis was excluded to avoid interference.

### 2.3 Serum Ferritin Measurement

Serum ferritin was measured using the Mouse Ferritin (FTL) ELISA Kit (Abcam, Concord, Canada; ab157713), following the manufacturer's instructions. Serum samples were diluted 1:40 in 1× assay diluent, and 100 μL of each standard (12.5–400 ng/mL), zero control, or diluted sample was added to designated wells in duplicate. The plate was incubated at room temperature for 60 min, washed four times with 1× wash buffer, and incubated with 100 μL of enzyme‐antibody conjugate for 10 min in the dark. After an additional wash, 100 μL of 3,3′,5,5′‐tetramethylbenzidine (TMB) substrate solution was added, followed by a 10‐min incubation. The reaction was stopped with 100 μL of stop solution, and absorbance was recorded at 450 nm using a Biotek Synergy HTX multimode plate reader.

### 2.4 Plasma H_2_S Measurement

Plasma H_2_S was measured using a Dionex Ultimate 3000 UHPLC system equipped with a diode array detector set to 660 nm (Thermo Scientific) as previously described [9]. Aliquots (20 μL) of prepared plasma samples were injected onto a Hypersil GOLD C18 column (250 × 4.6 mm, 5 μm), with a 20 min gradient elution of acetonitrile and 0.1% trifluoroacetic acid at a flow rate of 1 mL/min. NaHS standards were prepared by diluting stock solutions in 1% zinc acetate. N,N‐dimethyl‐p‐phenylenediamine (DPD) and FeCl₃ were subsequently added, and the mixture was incubated in the dark for 20 min. The reaction was then cleaned up by chloroform extraction, and the aqueous phase was mixed with methylene green as an internal standard. Samples were incubated overnight at 4°C to ensure complete methylene blue generation. H_2_S concentrations were determined from the methylene blue peak areas in the chromatograms.

Unless specified separately before, all chemicals used for H_2_S stabilization and detection were obtained from Sigma‐Aldrich (Oakville, Canada).

### 2.5 H_2_S Production Rate

The H_2_S production rate was assessed using a previously described lead acetate assay [4]. Briefly, lead acetate paper was prepared by soaking filter paper in 1% lead acetate solution for 1 h and drying it in an oven. Liver lysates (1% w/v) prepared in ice‐cold PBS (50 μL per well) were incubated with 10 μL of 10 mM cysteine and 10 μL of 2 mM pyridoxal 5′‐phosphate in a 96‐well plate, which was overlaid with the lead acetate paper. The plate was sealed with aluminum foil and incubated at 37°C for 2 h. The resulting darkened circular bands on the lead acetate paper, representing enzymatic H_2_S production, were imaged and analyzed using ImageJ software (version 1.54 h, NIH, Bethesda, MD, USA) [10].

Lead acetate, cysteine, pyridoxal 5′‐phosphate, and PBS components were all obtained from Sigma‐Aldrich (Oakville, Canada).

### 2.6 Histology and Immunofluorescence

Excised aortas were submerged in 10% neutral‐buffered formalin overnight, rinsed thoroughly with PBS, and embedded in Optimal Cutting Temperature (OCT) compound. Tissues were rapidly frozen in liquid nitrogen, stored at −80°C, and cryosectioned at 10 μm thickness using a Leica CM1850 UV microtome‐cryostat (Leica Biosystems, Concord, Canada). Sections were mounted onto Superfrost Plus slides (Fisherbrand, Ottawa, Canada) and incubated at 37°C for 30 min to enhance tissue adhesion. Before staining, slides were equilibrated to room temperature and rinsed with PBS to remove residual OCT.

For Prussian blue staining, tissue sections were incubated with freshly prepared Prussian blue solution (2% potassium ferrocyanide and 2% hydrochloric acid, 1:1) for 20 min at room temperature, rinsed thoroughly with distilled water, and counterstained with nuclear fast red for 5 min. After additional rinsing, sections were dehydrated through graded ethanol, cleared with xylene, and mounted in Permount (Fisher Scientific, Ottawa, Canada). Images were acquired using an Olympus X71 fluorescence microscope (Olympus America Inc., Center Valley, PA, USA), and the percentage of positive staining area was quantified using ImageJ.

EVG staining was performed using in‐house–prepared reagents. The working elastin solution (Verhoeff's hematoxylin) was prepared by mixing 20 mL of 5% alcoholic hematoxylin, 8 mL of 10% ferric chloride, and 8 mL of Lugol's iodine. Tissue sections were stained in this solution for 10 min, rinsed with deionized water, and differentiated in 2% ferric chloride solution for up to 2 min with microscopic monitoring. After rinsing in tap water for 5 min, sections were treated with 95% alcohol to remove residual iodine and rinsed again with deionized water. Counterstaining was performed for 1–3 min in Van Gieson's solution (5 mL of 1% acid fuchsin in 95 mL saturated picric acid). Finally, sections were rinsed in 95% alcohol, dehydrated, cleared in xylene, and mounted in Permount. Images were acquired using an Olympus X71 fluorescence microscope, and the percentage of positive staining area was quantified using ImageJ.

Aortic cryosections were blocked with 5% bovine serum albumin (BSA) in Tris‐buffered saline containing 0.1% Tween‐20 (TBST) for 1 h at room temperature and incubated overnight at 4°C in humidified chambers with primary antibodies: CD68 (1:200; Cell Signaling Technology, Whitby, ON, Canada), α‐Smooth Muscle Actin (αSMA, 1:400; Alexa Fluor 488 Conjugate; Cell Signaling Technology, Whitby, ON, Canada), or MMP9 (1:200; Thermo Fisher Scientific, Mississauga, ON, Canada). After washing with TBST, sections were incubated for 1 h at room temperature with Alexa Fluor 488 Goat anti‐Rabbit IgG (H + L) (1:200; Thermo Fisher Scientific, Mississauga, ON, Canada) or Alexa Fluor 594 Goat anti‐Rabbit IgG (H + L) (1:400; Thermo Fisher Scientific, Mississauga, ON, Canada), as appropriate. Sections were mounted with 10% glycerol in PBS containing DAPI (1 μg/mL) and covered with coverslips. Imaging was performed using an Olympus X71 fluorescence microscope (Olympus America Inc., Center Valley, PA, USA) with DAPI, FITC, and TRITC channels, and mean fluorescence intensity was quantified using ImageJ.

All chemicals used for Prussian blue and EVG staining, including potassium ferrocyanide, ferric chloride, hydrochloric acid, picric acid, and acid fuchsin, were obtained from Sigma‐Aldrich (Oakville, Canada). Tween‐20 was obtained from Fisher Chemicals (Mississauga, Canada).

### 2.7 Measurement of Isometric Tension of Vascular Tissues

Isometric tension was measured using our previously defined method [11]. Thoracic aortas from treated mice were excised and immediately placed in ice‐cold modified Krebs bicarbonate solution (in mM: 115 NaCl, 5.4 KCl, 2.5 CaCl_2_, 1.2 MgSO_4_, 1.2 KH_2_PO_4_, 25 NaHCO_3_, 5 D‐glucose, and 10 HEPES; pH 7.4) continuously aerated with 5% CO_2_ and 95% O_2_. Under a dissection microscope, all extraneous fat and connective tissue were carefully removed. Aortic rings (3–4 mm) were mounted on 0.22 mm stainless steel wires (Harvard Apparatus, Saint‐Laurent, Canada) connected to a force transducer (AD Instruments, Mississauga, Canada) and organ bath (Harvard Apparatus, Saint‐Laurent, Canada) maintained at 37°C with continuous aeration. Isometric tension was recorded using LabChart 8 software (AD Instruments, Mississauga, Canada).

Danish MyoTechnologies (DMT) normalization was used to ensure vessels were set at their optimal passive tension, corresponding to the optimal length–tension relationship for smooth muscle contraction. This approach enhances reproducibility and comparability across vessels and experiments [12]. Aortic rings were incrementally stretched between 1 and 4 mN and challenged with 60 mM KCl, with maximum active tension recorded. After three KCl challenges, the DMT curve was plotted using the DMT module in LabChart 8, and optimal passive tension was established. A fourth KCl challenge confirmed this setting, followed by three washes (5‐min intervals) and a 30‐min equilibration period (with a buffer exchange at 15 min).

Contractile responses were assessed by a single‐concentration challenge with 1 μM phenylephrine (PE), followed by relaxation with 10 μM acetylcholine (ACh). After four washes (5‐min intervals), rings were incubated with 0.1 mM Nω‐Nitro‐L‐arginine methyl ester hydrochloride (L‐NAME; Sigma‐Aldrich, Oakville, Canada) for 15 min to inhibit endothelial nitric oxide synthase, then challenged with 1 μM PE and relaxed with 10 μM hydrogen sulfide (H_2_S), prepared by diluting a 0.8 M stock solution (Sigma‐Aldrich, Oakville, Canada) in PBS. After washing and 15 min of re‐equilibration, a final 1 μM PE challenge was performed, followed by relaxation with 10 μM sodium nitroprusside (SNP) to evaluate smooth muscle integrity. Contraction was defined as the increase in force from baseline following PE addition, and relaxation was expressed as the percentage decrease in force relative to the maximum stable contraction induced by PE. All pharmacological agents (PE, ACh, SNP, H_2_S) and the salts used to prepare the Krebs bicarbonate solution were purchased from Sigma‐Aldrich (Oakville, Canada).

### 2.8 Blood Pressure Measurement

Systolic blood pressure (SBP) and diastolic blood pressure (DBP) were assessed using a noninvasive tail‐cuff system (CODA‐6, Kent Scientific, Torrington, CT, USA) as previously described [13]. Mice were acclimated to the restrainers and tail‐cuff apparatus for 2–3 sessions before measurements to minimize stress‐related artifacts. For each animal, more than six consecutive readings were recorded, and the mean value was used as the final measurement.

### 2.9 Western Blotting

For aortic samples, three thoracic aortas were pooled, pulverized into powder in liquid nitrogen, and lysed in ice‐cold lysis buffer (1× RIPA supplemented with 1% Phenylmethylsulfonyl fluoride and 1% protease inhibitor cocktail). Liver samples from individual mice (1% w/v) were also homogenized in ice‐cold lysis buffer. Protein lysates were resolved on 7.5% or 10% SDS‐PAGE gels and transferred onto PVDF membranes. Membranes were blocked with 5% non‐fat milk in TBST for 1 h at room temperature and incubated overnight at 4°C with primary antibodies: CSE (1:1000; Proteintech, San Diego, CA, USA), ferritin (1:2000; Novus Biologicals, Etobicoke, ON, Canada), GAPDH (1:2000; Novus Biologicals, Etobicoke, ON, Canada), DMT1 (1:1000; Thermo Fisher Scientific, Mississauga, ON, Canada), transferrin receptor 1 (TfR1, 1:1000; Thermo Fisher Scientific, Mississauga, ON, Canada), and ferroportin (FPN, 1:1000; Thermo Fisher Scientific, Mississauga, ON, Canada). After washing with TBST, membranes were incubated with HRP‐conjugated goat anti‐rabbit secondary antibody (1:2000; Thermo Fisher Scientific, Mississauga, ON, Canada) for 1.5 h at room temperature. Detection was performed using ECL (Cytiva, Montreal, QC, Canada) or Super Signal West Pico Plus (Thermo Fisher Scientific, Mississauga, ON, Canada), and images were captured using a Microchemi imaging system (Froggabio, Concord, ON, Canada). GAPDH was used as the loading control, and each blot was performed in triplicate to ensure reproducibility.

All other general chemicals used for lysis and washing (e.g., RIPA buffer components, PMSF, and protease inhibitor cocktail) were obtained from Sigma‐Aldrich (Oakville, Canada).

### 2.10 Quantitative PCR

Total RNA was isolated from 1% liver homogenates using TRIzol reagent (Sigma‐Aldrich, Oakville, ON, Canada), following the manufacturer's protocol. Reverse transcription was performed with 1 μg of total RNA using the Superscript First‐Strand cDNA Synthesis System (Invitrogen, Burlington, ON, Canada). Quantitative PCR (qPCR) was conducted using SYBR Green PCR Master Mix (BioRad, Mississauga, ON, Canada) on an iCycler iQ5 (BioRad) with optical system software (version 3.1). Hepcidin primers were forward: GCACCACCTATCTCCATCAACA and reverse: TTCTTCCCCGTGCAAAGG. The endogenous reference gene was 18S rRNA (forward: AGTCCCTGCCCTTTGTACACA, reverse: CGATCCGAGGGCCTCACTA).

The cycling program consisted of an initial denaturation at 95°C for 1 min, followed by 38 cycles of 95°C for 15 s, 58°C for 20 s, and 72°C for 30 s, with real‐time data acquisition during the extension step. A standard melt curve was performed from 58°C to 95°C in 1°C increments to confirm amplification specificity. Relative mRNA levels were calculated using the 2^−^ΔΔCT method, with ΔCT representing the difference between the target gene and the 18S reference threshold cycle [1].

### 2.11 Statistical Analysis and Reproducibility

All data are presented as means ± standard error (SEM). Unless specified otherwise, individual data points for the mean value of each experimental group were included, representing biological replicates (or the average of technical replicates where applicable). For plasma, serum, blood pressure, liver qPCR, and H_2_S production rate assays, *n* represents the number of mice (6–8 per group). For Western blotting, each biological replicate consisted of a pooled sample of three thoracic aortas (*n* = 1), with six independent pooled samples prepared per group; each sample was run in duplicate across three separate gels, and band intensities were averaged for analysis. For histological analyses (Prussian blue, EVG, IF), three randomly selected fields per section were quantified and averaged to generate one value per mouse. For experiments involving two factors (genotype and iron loading concentration), two‐way ANOVA was used to evaluate the main effects and their interaction, followed by Tukey's post hoc test where appropriate, whereas one‐way ANOVA was applied in cases where one factor showed no measurable values or changes (e.g., non‐detectable expression or no visible activity). A *p*‐value < 0.05 was considered statistically significant. A complete summary of tests, *F*‐statistics, degrees of freedom, and *p*‐values is provided in Table S1.

## 3 Results

### 3.1 CSE Deficiency Impairs Systemic Iron Homeostasis

**FIGURE 1:**
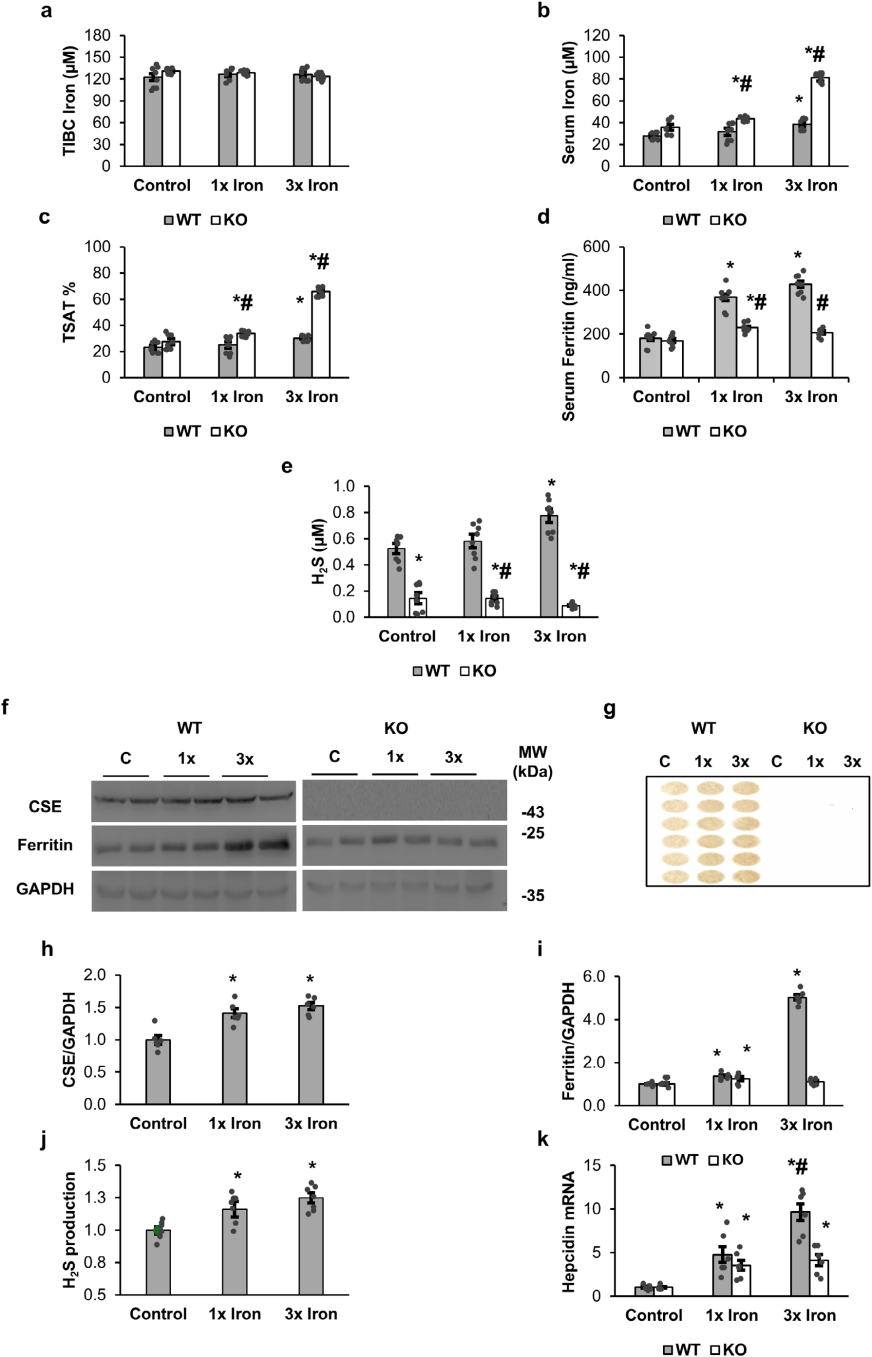
H_2_S and iron regulation parameters in plasma and liver following acute iron loading in WT and CSE‐KO mice. (a) Total iron‐binding capacity (TIBC) in serum. (b) Serum iron levels. (c) Transferrin saturation (TSAT) calculated from serum iron and TIBC. (d) Serum ferritin concentrations. (e) Plasma H_2_S levels measured by the MBMG method. (f) Representative Western blots of hepatic CSE, ferritin, and GAPDH. (g) Representative images of hepatic H_2_S production rates assessed by the lead acetate assay. (h) Relative hepatic CSE expression normalized to GAPDH. (i) Relative hepatic ferritin expression normalized to GAPDH. (j) Hepatic H_2_S production rates normalized to control. (k) Hepatic hepcidin mRNA levels relative to 18S rRNA. Effects of mouse genotypes, iron loading concentrations, and their interaction were assessed by two‐way ANOVA. Significance symbols represent Tukey's post hoc comparisons. *p < 0.05 versus WT control; #*p* < 0.05 versus WT group with the same iron treatment condition. *n* = 6 per group for Western blot and H_2_S production rate; *n* = 8 per group for the measurement of TIBC, serum iron, TSAT, serum ferritin, and plasma H_2_S.

The Total iron‐binding capacity (TIBC) remained unchanged between control, 1× iron, and 3× iron groups for both WT and KO (Figure 1a). In contrast, serum iron level increased significantly with iron loading, with a stronger rise in KO than WT (Figure 1b). In WT, serum iron levels increased moderately only with 3× iron (38.4 μM vs. 28.0 μM in controls), whereas KO serum iron doubled (81.4 μM vs. 35.8 μM in controls). Transferrin saturation (%TSAT) mirrored serum iron levels, rising to 65.9% in KO 3× iron compared to 27.4% in KO controls (Figure 1c). Serum ferritin levels increased markedly in WT (181 ng/mL in controls to 429 ng/mL with 3× iron), while serum ferritin levels in KO showed only a mild change (Figure 1d). Plasma H_2_S levels increased in WT following iron injections (0.52 μM in controls to 0.77 μM in 3× iron), whereas KO exhibited consistently low levels with no iron‐induced changes (Figure 1e).

Representative liver blots (Figure 1f) showed that CSE and ferritin proteins were upregulated in WT, while KO displayed no detectable CSE. Quantification confirmed significant induction of liver CSE and ferritin with iron in WT (5‐fold ferritin increase at 3× iron) but not in KO (Figure 1h,i). H_2_S production rates were elevated in WT with iron loading but remained undetectable in KO (Figure 1j,g). Hepcidin expression increased after 1× iron in both genotypes but rose significantly higher in WT than KO with 3× iron (9.5‐fold vs. 4.9‐fold) (Figure 1k).

### 3.2 CSE Deficiency Impairs Vascular Iron Homeostasis

**FIGURE 2:**
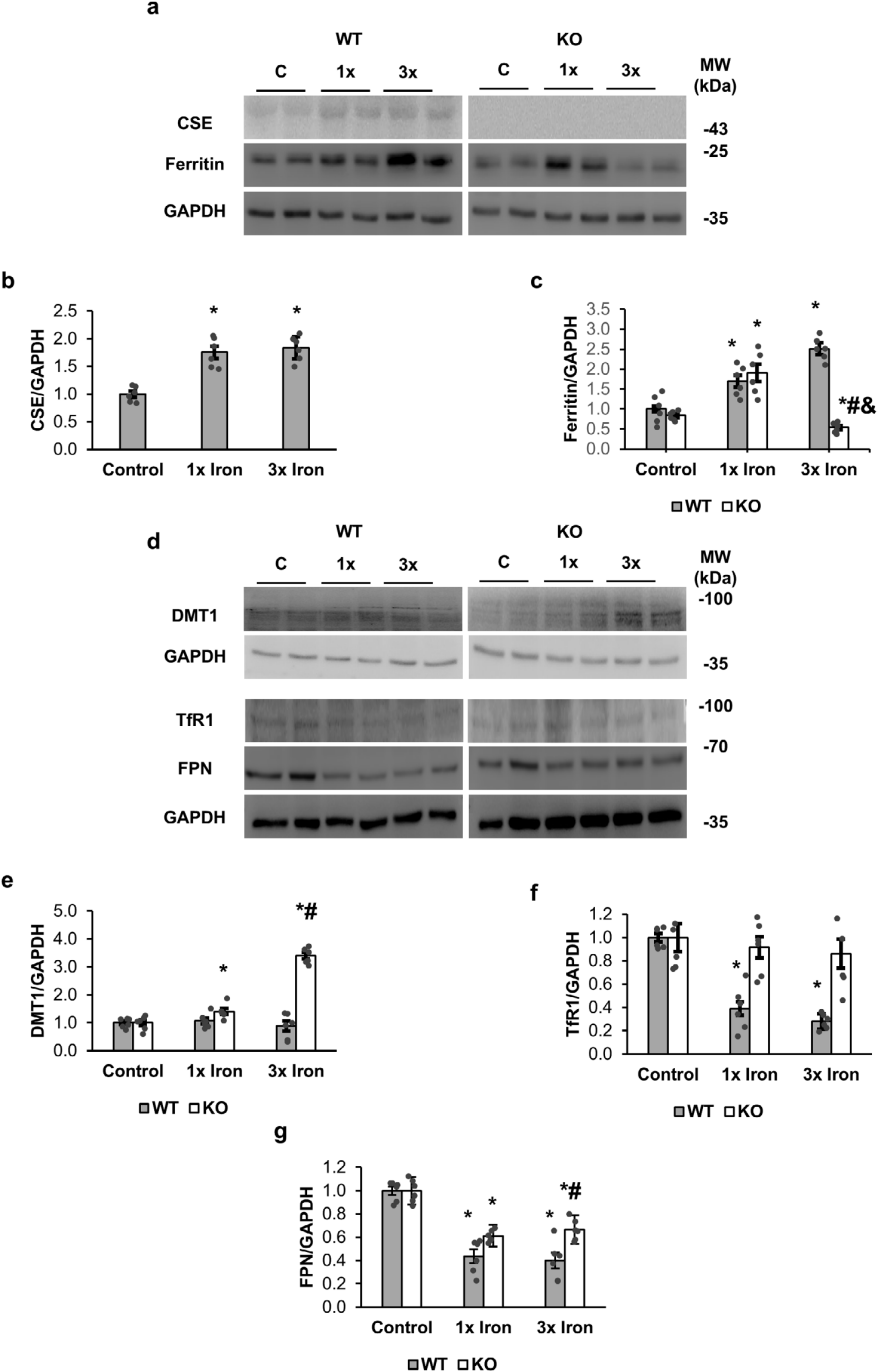
Aortic expression of CSE, ferritin, and iron transporters following acute iron loading in WT and CSE‐KO mice. (a) Representative Western blots of aortic CSE, ferritin, and GAPDH. (b) Relative expression levels of aortic CSE normalized to GAPDH. (c) Relative expression levels of aortic ferritin proteins normalized to GAPDH. (d) Representative Western blots of DMT1, transferrin receptor 1 (TfR1), ferroportin (FPN), and GAPDH. (e) Relative expression levels of DMT1 normalized to GAPDH. (f) Relative expression levels of TfR1 normalized to GAPDH. (g) Relative expression levels of FPN normalized to GAPDH. Effects of mouse genotypes, iron loading concentrations, and their interaction were assessed by two‐way ANOVA. Significance symbols represent Tukey's post hoc comparisons. *p < 0.05 versus WT control; #*p* < 0.05 versus WT group at the same iron concentration; and *p* < 0.05 versus KO control. *n* = 6.

Aortic CSE protein levels increased with iron loading in WT but were absent in KO (Figure 2a,b). Ferritin expression levels increased in both genotypes with 1× iron; however, with 3× iron, WT ferritin increased 2.5‐fold, while KO ferritin dropped to 0.53‐fold of the control levels (Figure 2a,c). DMT1 and TfR1 mediate cellular iron uptake, whereas FPN functions as the iron exporter, and their expression patterns are shown in Figure 2d–g. DMT1 remained stable in WT aortic tissues, but DMT1 increased nearly 3‐fold in KO aortic tissues with 3× iron (Figure 2e), while TfR1 progressively decreased in WT with iron loading (to 28% of control levels at 3× iron) but showed minimal reduction in KO (Figure 2f). FPN also decreased in both genotypes, with a greater reduction in WT (Figure 2g).

### 3.3 Iron Deposition and Vascular Remodeling Are Exacerbated in KO

**FIGURE 3:**
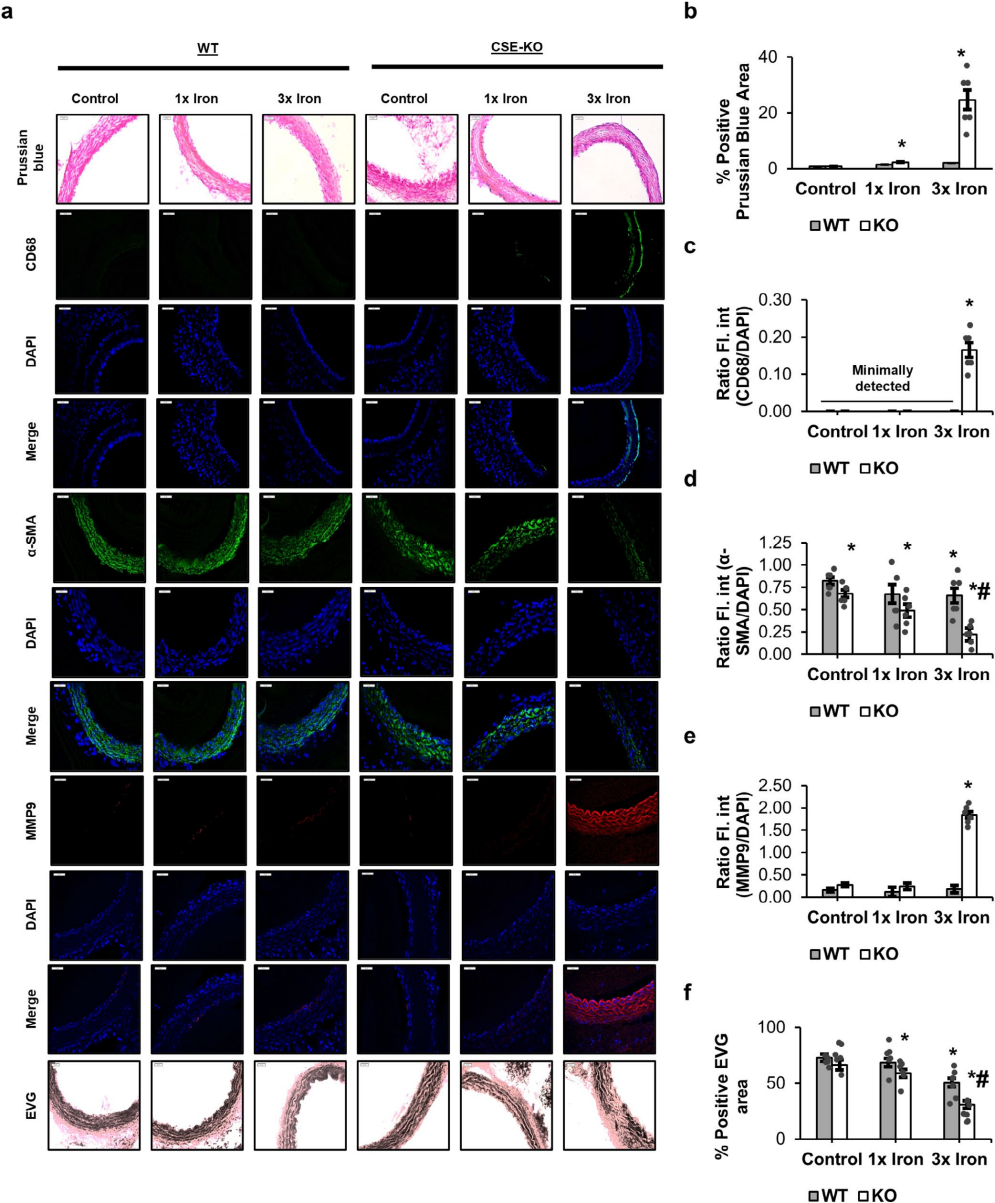
Histological and immunofluorescence analysis of vascular remodeling following acute iron loading in WT and CSE‐KO mice. (a) Representative images of Prussian blue staining, CD68, αSMA, MMP9, and EVG staining in aortic sections. (b) Quantification of Prussian blue–positive area. (c) Mean fluorescence intensity ratio of CD68 to DAPI. (d) Mean fluorescence intensity ratio of αSMA to DAPI. (e) Mean fluorescence intensity ratio of MMP9 to DAPI. (f) Percentage of EVG‐positive elastin content. Effects of mouse genotypes, iron loading concentrations, and their interaction were assessed by two‐way ANOVA. Significance symbols represent Tukey's post hoc comparisons. *p < 0.05 versus WT control; #*p* < 0.05 versus WT group at the same iron concentration. *n* = 6 per treatment group.

Prussian blue staining revealed significant iron deposits only in vascular tissues from KO mice with 3× iron treatment (Figure 3a,b). CD68‐positive macrophage infiltration was also observed exclusively in the 3× iron group of KO mice (Figure 3c). αSMA staining, a marker of smooth muscle integrity, decreased greatly in the 3× iron group of KO mice with iron loading (0.22 ± 0.11 αSMA/DAPI ratio vs. 0.67 ± 0.12 in KO controls) but showed only a mild and insignificant reduction in WT (Figure 3d). MMP9 expression increased significantly only in KO mice with 3× iron treatment (Figure 3e). The percentage of EVG‐positive staining decreased by ~2.1‐fold in KO vascular tissues (from 66% positive stain area in controls to 31% with 3× iron), whereas WT vascular tissues showed ~1.5‐fold decrease (from 73% to 50%) (Figure 3f).

High‐resolution images for Prussian blue and EVG staining are provided in Figures S1 and S2.

### 3.4 CSE Deficiency Increases Vascular Stiffness and Blood Pressure With Iron Loading

**FIGURE 4:**
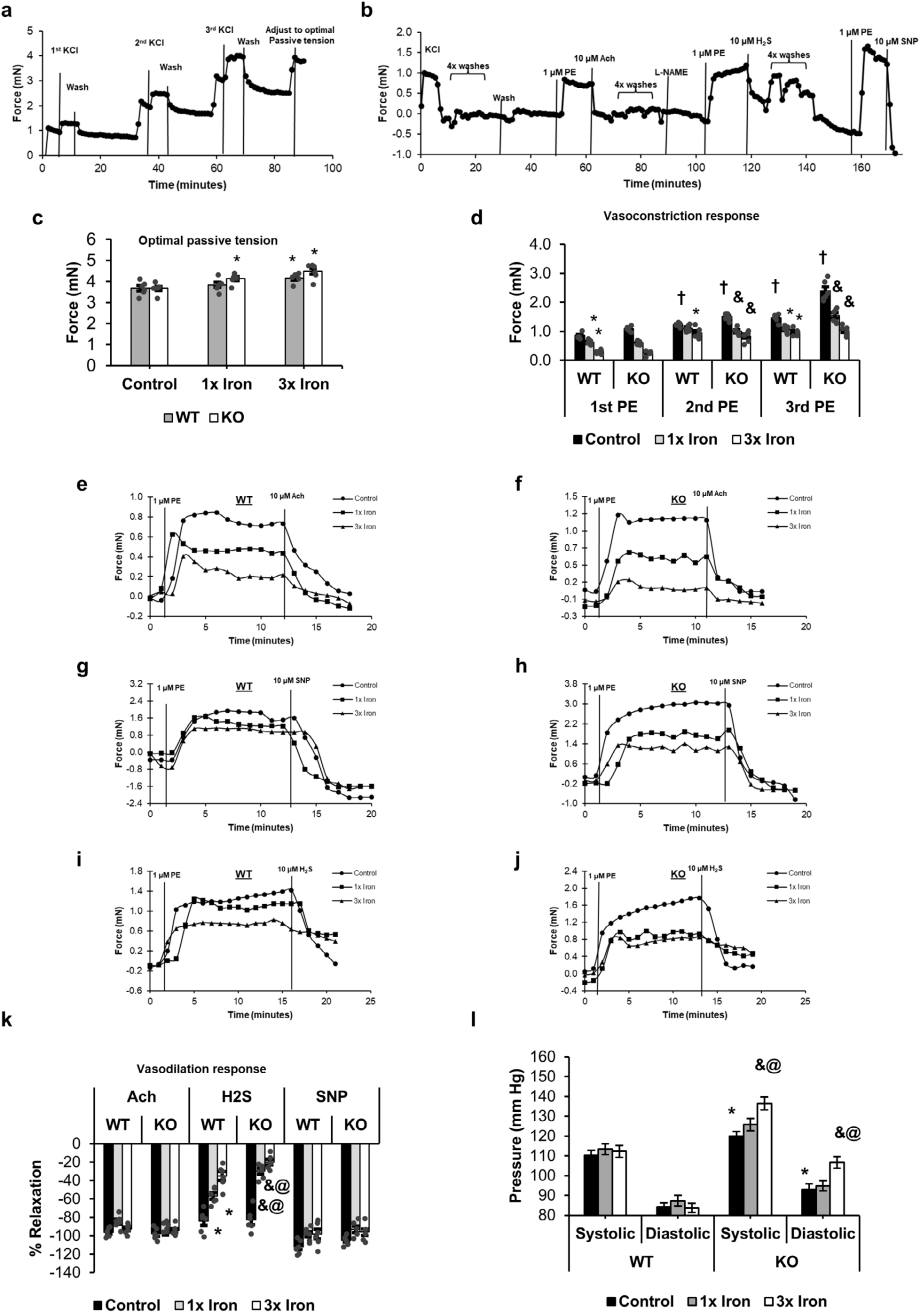
Vasomotor responses and blood pressure changes following acute iron loading in WT and CSE‐KO mice. (a) Time–force trace showing DMT normalization for determining optimal passive tension. (b) Representative recording of the contraction–relaxation sequence (PE, ACh, H_2_S, SNP) in aortic rings. (c) Optimal passive tension determined from DMT normalization. (d) Peak contractile responses to three consecutive PE challenges, with the first measured before L‐NAME treatment and the second and third measured after L‐NAME conditioning. (e, f) Snapshots of PE contraction followed by ACh‐induced relaxation in WT and KO aortae. (g, h) Snapshots of PE contraction followed by SNP‐induced relaxation in WT and KO aortae. (i, j) Snapshots of PE contraction followed by H_2_S‐induced relaxation in WT and KO aortae. (k) Summary of the vasorelaxation responses to ACh, H_2_S, and SNP. (l) Systolic and diastolic blood pressure measured via tail‐cuff method. Scattered individual points are not shown for the mean blood pressure means due to the high number of replicate readings per mouse. Effects of mouse genotypes, iron loading concentrations, and their interaction were assessed by two‐way ANOVA. Significance symbols represent Tukey's post hoc comparisons. *p < 0.05 versus WT control; &*p* < 0.05 versus KO control; †*p* < 0.05 versus the first PE response of the control group (same genotype); @*p* < 0.05 versus WT with the same iron treatment conditions. *n* = 6 with 2 replicates per treatment group for vascular tension studies and *n* = 8 per treatment group for blood pressure measurements.

A representative isometric tension development recording for determining optimal passive tension through DMT normalization is shown in Figure 4a. A representative original isometric recording of sequential applications of PE, ACh, L‐NAME, H_2_S, and SNP is shown in Figure 4b. Optimal passive tension increased in KO mice with 1× iron treatment (4.14 mN vs. 3.84 mN in WT tissues) and in both genotypes with 3× iron treatments, with KO tissues showing a modest (~8%) higher tension compared to WT tissues (Figure 4c). Representative complete traces are provided in Figure S3.

PE‐induced vasocontraction increased notably after L‐NAME conditioning (Figure 4d). The second PE response rose by ~43% in control WT tissues and ~38% in control KO tissues compared to the pre‐L‐NAME levels. The third PE response remained elevated at ~70% higher than pre‐L‐NAME level in WT and ~120% higher in KO tissues. This demonstrates that L‐NAME, by inhibiting endothelial NO production, increased PE‐induced contractions, providing a higher baseline tone.

Without L‐NAME treatment, iron loading caused a wide variation in PE‐induced contraction magnitudes among control, 1×, and 3× iron groups. For example, in WT aortic rings, contraction forces dropped by ~28% with 1× and ~64% with 3× iron compared to control. A similar but more pronounced decrease was seen in KO aortic rings (−45% with 1× iron and −76% with 3× iron). After L‐NAME conditioning (second and third PE stimulations), these group differences became less pronounced due to the overall increase in contractile force.

With L‐NAME treatment, the third PE‐induced contraction of KO tissues remained more sensitive to iron, with reductions of ~35% (1× iron) and ~54% (3× iron) compared to KO control. In contrast, WT aortic tissues exhibited smaller decreases of ~27% (1× iron) and ~33% (3× iron) (Figure S4). These findings confirm that vascular smooth muscle contractility is more sensitive to iron loading in the absence of CSE. This interpretation was supported by two‐way ANOVA, which revealed a significant interaction of mouse genotypes with iron loading concentrations for PE‐induced contractions.

ACh‐induced vasorelaxation was examined in the absence of L‐NAME treatment. With different baselines of PE‐induced contractions in aortic tissues, ACh relaxed all the tissues almost completely within 10 min for both WT tissues (Figure 4e) and KO tissues (Figure 4f). In the following experiments with H_2_S and SNP, the aortic rings were all pre‐treated with L‐NAME in order to block eNOS‐derived NO. SNP induced the same extents of vasorelaxation among all WT (Figure 4g) and KO aortic tissues (Figure 4h), confirming that the vasorelaxant responses of smooth muscles to the NO donor were not changed by iron treatments. In contrast, in WT aortae, H_2_S‐induced relaxation decreased by ~32% with 1× iron and ~58% with 3× iron compared to controls (Figure 4i). KO aortic tissues showed a greater loss, with reductions of ~64% (1× iron) and ~76% (3× iron) relative to controls (Figure 4j).

Different vasorelaxation responses were further quantified in Figure 4k as a percentage of the preceding PE‐induced contraction under each treatment condition, allowing comparison based on the same contractile baseline tone. It is clear that while ACh‐ and SNP‐induced vasorelaxations were not affected by iron treatments, H_2_S‐induced vasorelaxation was severely decreased by iron treatment and more so in KO vascular tissues. Two‐way ANOVA revealed significant main effects of both mouse genotype (WT vs. KO) and iron loading concentrations (control, 1×, 3×), as well as a significant interaction of mouse genotypes and iron loading concentrations. This means that while mouse genotypes and iron loading concentrations each independently influenced the extent of H_2_S‐induced vasorelaxation, the decrease in vasorelaxation was disproportionately greater in KO tissues with iron loading.

WT mice at the age of 8 weeks had stable systolic and diastolic pressures, which were not significantly affected by iron treatments. In contrast, KO mice of the same age exhibited significant increases in both SBP and DBP with 3× iron loading (> 10% above control). Two‐way ANOVA confirmed main effects of genotype (WT vs. KO) and iron concentration (control, 1×, 3×), as well as a significant genotype × concentration interaction (SBP: Fgenotype(1,42) = 2151.8, Fconcentration(2,42) = 416.8, Finteraction(2,42) = 147.3; DBP: Fgenotype(1,42) = 37.9, Fconcentration(2,42) = 3.2, Finteraction(2,42) = 5.4; all *p* < 0.05). These results demonstrate that while both genotype and iron concentration independently influence blood pressure, the hypertensive effect of iron loading is disproportionately greater in KO mice, whereas WT mice maintained relatively stable pressures across treatments.

## 4 Discussion

Our study reveals a previously unrecognized role of the CSE‐derived H_2_S in systemic and vascular iron regulation, showing that CSE deficiency leads to impaired ferritin upregulation, abnormal iron accumulation, and pronounced vascular remodeling following acute iron loading. CSE‐KO mice exhibited elevated serum iron and transferrin saturation with a blunted hepcidin response, accompanied by a decreased ferritin induction compared to WT. This suggests that CSE‐KO mice fail to effectively sequester excess iron in as ferritin, which is a redox‐inactive storage form [14]. In iron‐treated KO aortas, ferroportin failed to be downregulated, and DMT1 expression was elevated. Combined with impaired ferritin upregulation, this leads to labile iron accumulation and oxidative stress. The increase in DMT1 expression in KO mice is likely due to impaired IRP1‐to‐aconitase conversion, leaving more active IRP1 bound to the DMT1 iron response elements (IRE) in DMT1 mRNA. With high iron loading, IRP1 should convert to aconitase and detach from DMT1 mRNA as occurred in WT tissues, causing its breakdown [15]. In CSE‐KO mice, this regulation fails due to impaired IRP1‐to‐aconitase conversion [4, 5] resulting in stable DMT1 expression [15]. These findings are consistent with our earlier cell‐based study [4], where H_2_S deficiency in VSMCs disrupted IRP1‐to‐aconitase conversion, resulting in low ferritin expression and increased iron accumulation. Zhu et al. similarly demonstrated that H_2_S stabilizes iron homeostasis through IRP1/IRE interactions [5]. Extending these in vitro results, our in vivo data show for the first time that tail‐vein iron injection in WT mice upregulates ferritin expression in both livers and aortae, while KO mice fail to mount a comparable response, particularly with high‐iron loading conditions.

CSE expression in liver and aortic tissues was also elevated by iron loading in WT mice, in line with our previous finding that iron upregulates CSE through the integrated stress response via ATF4 [4]. The associated rise in plasma H_2_S levels and hepatic H_2_S production in WT mice likely reflects compensatory activation of liver CSE, a major H_2_S‐producing tissue [3]. In contrast, iron‐loaded KO mice exhibited persistently low plasma H_2_S, correlating with impaired vasorelaxant responses and systemic iron dysregulation. Histological results support the idea that impaired ferritin upregulation enhances iron toxicity, as shown by strong Prussian blue staining in KO aortae after 3× iron loading, despite lower ferritin. This parallels observations in H‐ferritin knockout mice, where the absence of functional ferritin led to an increase in labile iron pools and significant iron accumulation in the spleen that was observable via Prussian blue staining [16]. Our findings suggest that the failure to upregulate ferritin in KO mice keeps iron in reactive, labile forms rather than safely stored as ferritin, amplifying oxidative injury in vascular tissues.

Vascular remodeling has been observed in KO mice, including loss of elastin (EVG), reduced αSMA, and increased CD68 and MMP9 staining, which reflect oxidative stress and inflammation [4]. Previous studies also report that immune activation caused by vascular oxidative stress leads to fibrosis and elevated blood pressure [17]. Moreover, Martínez‐Revelles et al. showed that lysyl oxidase‐mediated oxidative stress alters elastin structure and increases vascular stiffness via p38MAPK activation [18]. These structural changes are linked to vascular functional changes observed in our current study. Phenylephrine‐induced contraction was decreased with iron loading, particularly in CSE‐KO mice, reflecting smooth muscle depletion. This is consistent with findings by Kuang et al., who demonstrated that ferric ammonium citrate impairs PE‐induced vasoconstriction of rat aortic tissues, an effect reversed by antioxidants [19]. H_2_S‐mediated vasorelaxation was markedly decreased in iron‐loaded CSE‐KO mice compared to WT, an effect driven by iron‐induced ROS production that was further exacerbated in KO mice due to its inherently elevated oxidative stress. ROS can inhibit vascular K_ATP_ channels in the mesenteric artery via S‐glutathionylation [20], and our previous study confirmed that KO VSMCs generated substantially more ROS than WT cells when exposed to physiologically relevant concentrations of iron [4]. Since H_2_S‐induced vasorelaxation depends on K_ATP_ channel activation [21, 22], ROS‐induced inhibition of K_ATP_ channels in KO cells may account for the greater loss of the vasorelaxant effects of H_2_S in these cells with iron overloading.

Interestingly, we did not observe any significant changes in SNP‐mediated relaxation among all groups. SNP, as an NO donor, promotes vasorelaxation through activation of soluble guanylate cyclase (sGC), leading to increased intracellular cGMP levels and subsequent activation of protein kinase G (PKG), which decreases vascular smooth muscle tone [23]. Chronic oxidative stress is known to impair the NO–cGMP signaling pathway by suppressing PKG activation and inhibiting vasodilator‐stimulated phosphoprotein (VASP) phosphorylation, as shown in a study where no significant difference in cGMP‐dependent relaxation was observed after 1 week of oxidative stress in rat mesenteric artery, but a marked decrease occurred after 3 weeks [24]. In contrast, our acute model (24 h post‐iron loading) revealed a decrease in H_2_S‐mediated relaxation, suggesting that the H_2_S signaling pathway is more vulnerable to early oxidative perturbations than the NO–cGMP pathway [25]. It is also possible that the relatively high concentrations of NO generated by SNP are sufficient to overcome mild acute oxidative inhibition of sGC. Future studies using chronic iron‐loading models will be necessary to determine whether prolonged oxidative stress eventually impairs NO–cGMP signaling in this context.

The impaired vasorelaxation, inflammatory remodeling (increased CD68 and MMP9), diminished contractile reserve due to smooth muscle depletion (decreased amount of αSMA), and significant elastin loss (EVG staining) are indicative of a maladaptive shift toward vascular stiffness and elevated tone, consistent with studies linking oxidative stress and impaired K_ATP_ channel function to hypertensive pathology [17, 26]. Furthermore, vascular functional deterioration observed in our current study was accompanied by a rise in systolic and diastolic pressures in KO mice with high iron loading. In line with this, Sangartit et al. also reported hypertension and vascular dysfunction in iron‐overloaded mice [27].

Patients with conditions such as hereditary hemochromatosis, transfusion‐related iron overload, or those undergoing repeated intravenous iron therapy (e.g., in chronic kidney disease or thalassemia) often develop endothelial dysfunction and vascular complications due to excess free iron and oxidative stress [28]. The acute iron surges modeled by tail‐vein iron injections in our study parallel the vascular stress observed in these clinical settings. In KO mice, FPN levels failed to decrease as expected under iron loading, allowing iron to be exported from vascular tissues back into circulation rather than being sequestered as ferritin. This abnormal iron handling increases labile iron pools, exacerbating oxidative vascular damage. CSE‐KO mice display a unique vascular phenotype after acute iron loading, characterized by iron deposition, oxidative remodeling, attenuated PE constriction, and compromised H_2_S vasorelaxation. These findings position H_2_S deficiency as a mechanistically distinct pathway in vascular iron dysregulation, with direct clinical implications for conditions where impaired H_2_S signaling may amplify iron‐induced vascular injury, highlighting it as a potential therapeutic target.

In addition, our data may provide mechanistic insight into vascular complications observed in chronic kidney disease (CKD) and diabetes, where both H_2_S levels and vascular function are often impaired [29, 30]. Clinical studies have shown that weakened H_2_S signaling in CKD patients correlates with endothelial dysfunction and increased cardiovascular risk [31], which can be exacerbated by intravenous iron therapy [32]. In diabetes, iron overload is increasingly recognized as a comorbid factor contributing to vascular damage and metabolic dysfunction, as supported by meta‐analyses linking altered iron metabolism to type 2 diabetes [33]. Similarly, in diabetes, low H_2_S bioavailability has been linked to impaired vasorelaxation and oxidative stress in which STZ‐induced diabetic rats exhibited both lowered physiological tissue H_2_S levels and impaired endothelium‐dependent relaxation [34]. Our findings suggest that restoring H_2_S signaling in such conditions could mitigate the vascular toxicity of iron therapy, offering a potential therapeutic strategy.

While the current study focuses on iron overload, the relationship between H_2_S and anemia remains underexplored and warrants investigation. Since H_2_S modulates key iron metabolism pathways (including ferritin and transferrin receptor regulation), its deficiency could impair both iron mobilization during anemia and influence erythropoietic processes. This potential dual role is supported by existing evidence: CSE deficiency has been shown to increase erythropoiesis through upregulation of coproporphyrinogen III oxidase and stimulation of heme biosynthesis—a compensatory mechanism to enhance hemoglobin production under low H_2_S conditions [35]. Furthermore, decreased endogenous H_2_S production is linked to impaired renal erythropoietin synthesis [36], suggesting H_2_S may regulate erythropoiesis at multiple levels. These findings raise the possibility that CSE/H_2_S signaling serves as a critical regulator balancing iron availability in both deficiency and excess states.

The current study examines acute iron loading effects (24 h post‐injection), but chronic models will be essential to determine whether these early alterations progress to sustained vascular dysfunction, fibrosis, or hypertension. Future investigations should explore whether H_2_S's role in iron metabolism extends to influencing red blood cell quality and systemic iron homeostasis in anemia, potentially revealing novel therapeutic targets for iron‐related disorders.

## 5 Conclusion and Perspectives

This study reveals that CSE/H_2_S deficiency impairs iron homeostasis, leading to vascular iron accumulation, oxidative stress, and diminished H_2_S‐mediated vasorelaxation, ultimately contributing to early hypertensive changes under acute iron overload. These findings underscore the critical role of H_2_S as a protective factor against iron‐induced vascular injury. Future research should explore chronic iron‐loading models, sex‐specific responses, and the broader impact of H_2_S in other organs, including the heart, brain, and erythropoietic system. Therapeutic strategies aimed at restoring H_2_S signaling—such as exogenous donors (e.g., slow‐release H_2_S hydrogels [37]) or interventions boosting endogenous H_2_S production (e.g., metformin or dietary restriction [38, 39, 40, 41])—may mitigate oxidative stress, improve iron regulation, and prevent vascular dysfunction. Investigating these approaches, particularly in chronic settings, could offer translational insights for reducing cardiovascular risk in patients undergoing iron therapy or with H_2_S metabolism disorders, while also informing therapeutic strategies for anemia‐related iron dysregulation.

## Author Contributions

H.M.A.: conceptualization, methodology, data analysis, writing original draft. M.F.: methodology, data analysis. R.V.: methodology, R.W.: data analysis, writing, funding acquisition, project administration. All authors have read and agreed to the published version of the manuscript.

## Ethics Statement

The animal study protocol was approved by The Animal Care Committee, York University (2019‐11 R1).

## Conflicts of Interest

The authors declare no conflicts of interest.

## Supporting information


**Data S1:** fsb271162‐sup‐0001‐Supinfo.pdf.

## Data Availability

The original contributions presented in this study are included in the article. Further inquiries can be directed to the corresponding author.
