# Supplementary material for: CSE‐Derived Hydrogen Sulfide Safeguards Vascular Iron Homeostasis by Coupling Ferritin Buffering to Vasoprotection
Source: FASEB J. 2025 Oct 21;39(20):e71162. doi: 10.1096/fj.202502763RR (PMC12539386; doi:10.1096/fj.202502763RR)
Supplement: Supplementary file 1 — Data S1: fsb271162‐sup‐0001‐Supinfo.pdf. [file FSB2-39-e71162-s001.pdf]

Supplemental Information

CSE-derived hydrogen sulfide safeguards vascular iron homeostasis by coupling ferritin buffering to vasoprotection

Hassan Mustafa Arif [1], Ming Fu[1,2], Richa Verma[1], Rui Wang[1,2*]

1. Department of Biology, York University, Toronto, ON M3J 1P3, Canada; harif@yorku.ca (H.M.A.); mingf1@yorku.ca (M.F.); 24rverma24@gmail.com

2. College of Basic Medicine, Shandong Second Medical University, Weifang 261053, China

* To whom all correspondence should be addressed at ruiwang@yorku.ca

Keywords: Hydrogen sulfide, Iron overload, Vascular dysfunction, Oxidative stress, Hypertension

Running title: H_2S Protects Against Vascular Iron Overload

Supplementary Table 1. Summary of statistical analyses for all experimental outcomes presented in the main figures. The table lists the corresponding figure panels, measured variables, statistical tests performed, and the associated F-statistics (or equivalent test results) with degrees of freedom and p-values. Two-way ANOVA was used to assess the effects of genotype, treatment, and their interaction. In cases where one factor had no measurable values or changes (e.g., non-detectable expression or no visible production activity), one-way ANOVA was applied to evaluate treatment effects within the measurable group. Fgenotype = effect of genotype; Fconc = effect of iron concentration; Fint = genotype × concentration interaction.

Figure	Measured Variable	Statistical Test	F-Statistics
Fig.1b,	Serum iron	Two-way ANOVA	($F_{genotype}(1,42) = 182.3$; $F_{conc}(2,42) = 122.2$; $F_{int}(2,42) = 51.9$; all $p < 0.0001$)
Fig.1c	Tstat	Two-way ANOVA	($F_{genotype}(1,42) = 171.4$; $F_{conc}(2,42) = 128.5$; $F_{int}(2,42) = 62.3$; all $p < 0.0001$)
Fig. 1d	Serum Ferritin	Two-way ANOVA	($F_{genotype}(1,42) = 74.2$; $F_{conc}(2,42) = 141.5$; $F_{int}(2,42) = 34.5$; all $p < 0.0001$)
Fig.1e	Plasma H_2S	Two-way ANOVA	$F_{genotype}(1,42) = 322.3$; $F_{conc}(2,42) = 4.28$; $F_{int}(2,42) = 11.3$; all $p < 0.05$
Fig.1h	Liver CSE/GAPDH	One-way ANOVA	$F(2,15) = 18.85$, $p < 0.0001$
Fig.1i	Liver Ferritin/GAPDH	Two-way ANOVA	$F_{genotype}(1,30) = 417.5$; $F_{conc}(2,30) = 385.0$; $F_{int}(2,30) = 382.5$; all $p < 0.0001$
Fig.1j	Liver H_2S production rate	One-way ANOVA	$F(2,15) = 9.66$, $p = 0.0020$
Fig.1k	Liver Hepcidin	Two-way ANOVA	$F_{genotype}(1,30) = 15.6$; $F_{conc}(2,30) = 35.1$; $F_{int}(2,30) = 8.5$; all $p < 0.01$
Fig.2b	Aorta CSE/GAPDH	One-way ANOVA	$F(2,15) = 27.56$, $p < 0.0001$

Fig.2c	Aorta Ferritin/GAPDH	Two-way ANOVA	$F_{genotype}(1,30) = 34.7$; $F_{conc}(2,30) = 22.7$; $F_{int}(2,30) = 37.9$; all $p < 0.0001$
Fig.2e	Aorta DMT1/GAPDH	Two-way ANOVA	$F_{genotype}(1,30) = 98.5$; $F_{conc}(2,30) = 53.1$; $F_{int}(2,30) = 67.3$; all $p < 0.0001$
Fig.2f	Aorta TfR1/GAPDH	Two-way ANOVA	$F_{genotype}(1,30) = 13.6$; $F_{conc}(2,30) = 26.8$; $F_{int}(2,30) = 6.7$; all $p < 0.01$
Fig.2g	Aorta FPN/GAPDH	Two-way ANOVA	$F_{genotype}(1,30) = 15.6$; $F_{conc}(2,30) = 71.1$; $F_{int}(2,30) = 4.4$; all $p < 0.05$
Fig.3b	Prussian Blue	Two-way ANOVA	$F_{genotype}(1,30) = 39.7$; $F_{conc}(2,30) = 39.7$; $F_{int}(2,30) = 39.7$; all $p < 0.0001$
Fig.3c	CD68/DAPI	Two-way ANOVA	$F_{genotype}(1,30) = 59.9$; $F_{conc}(2,30) = 59.9$; $F_{int}(2,30) = 59.9$; all $p < 0.0001$
Fig.3d	α-SMA/DAPI	Two-way ANOVA	$F_{genotype}(1,30) = 23.7$; $F_{conc}(2,30) = 11.6$; $F_{int}(2,30) = 4.6$; $p < 0.05$
Fig.3e	MMP9/DAPI	Two-way ANOVA	$F_{genotype}(1,30) = 508.2$; $F_{conc}(2,30) = 508.2$; $F_{int}(2,30) = 508.2$; all $p < 0.0001$
Fig.3f	EVG	Two-way ANOVA	$F_{genotype}(1,30) = 11.9$; $F_{conc}(2,30) = 45.2$; $F_{int}(2,30) = 7.1$; all $p < 0.01$
Fig.4c	Optimal Pretension	Two-way ANOVA	$F_{genotype}(1,30) = 4.53$, $p < 0.05$; $F_{conc}(2,30) = 15.4$, $p < 0.0001$; $F_{int}(2,30) = 1.34$, ns
Fig.4d	Vasoconstriction response	Two-way ANOVA	$F_{genotype}(1,30) = 58.4$; $F_{conc}(2,30) = 60.9$; $F_{int}(2,30) = 11.6$; all $p < 0.001$

Fig.4k	H_2S Vasodilation response	Two-way ANOVA	$F_{genotype}(1,30) = 18.1$; $F_{conc}(2,30) = 95.3$; $F_{int}(2,30) = 4.8$; all $p < 0.05$
Fig.4l	Systolic	Two-way ANOVA	$F_{genotype}(1,42) = 2151.8$; $F_{conc}(2,42) = 416.8$; $F_{int}(2,42) = 147.3$; all $p < 0.0001$
Fig.4l	Diastolic	Two-way ANOVA	$F_{genotype}(1,42) = 37.9$, $p < 0.0001$; $F_{conc}(2,42) = 3.2$, $p = 0.05$; $F_{int}(2,42) = 5.4$, $p < 0.01$

Prussian Blue Staining

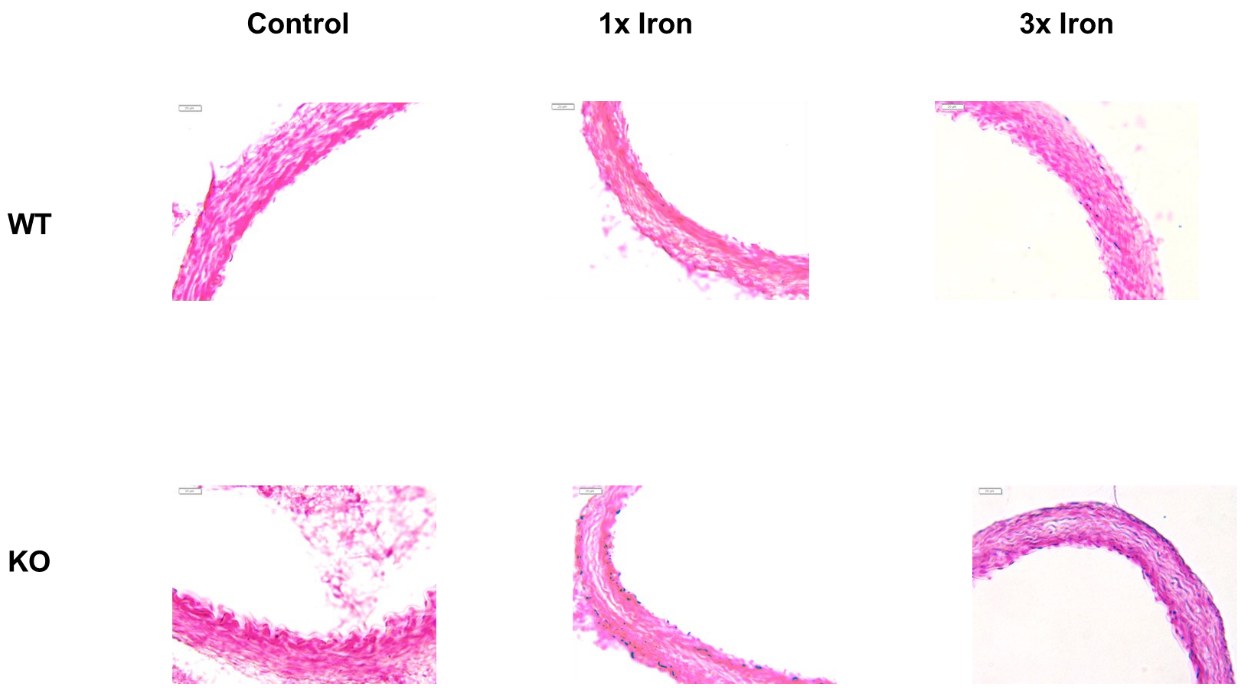

Supplementary Figure S1. Representative high-resolution images of Prussian Blue staining in aortic cross-sections from WT and CSE-KO mice following control, 1× iron, or 3× iron treatment. Scale bars = 20 μm.

EVG Staining

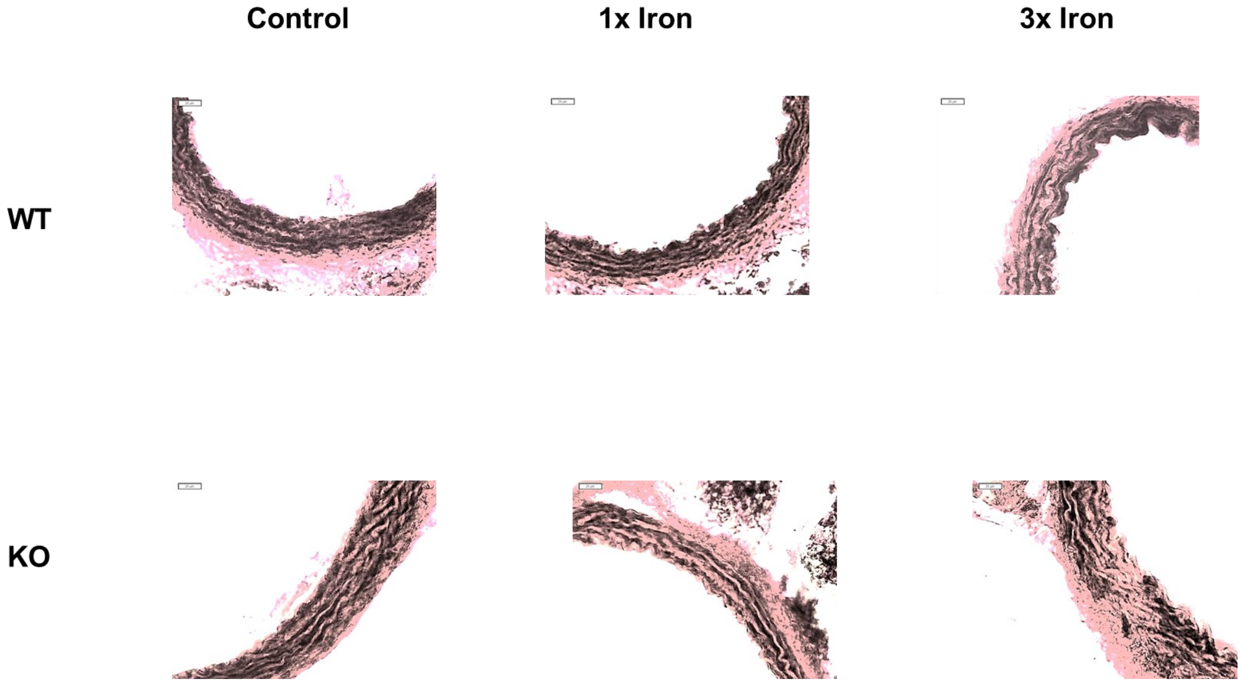

Supplementary Figure S2. High-resolution EVG-stained images of aortic cross-sections from WT and CSE-KO mice following acute iron loading (Control, 1× iron, 3× iron). Scale bars = 20 μm.

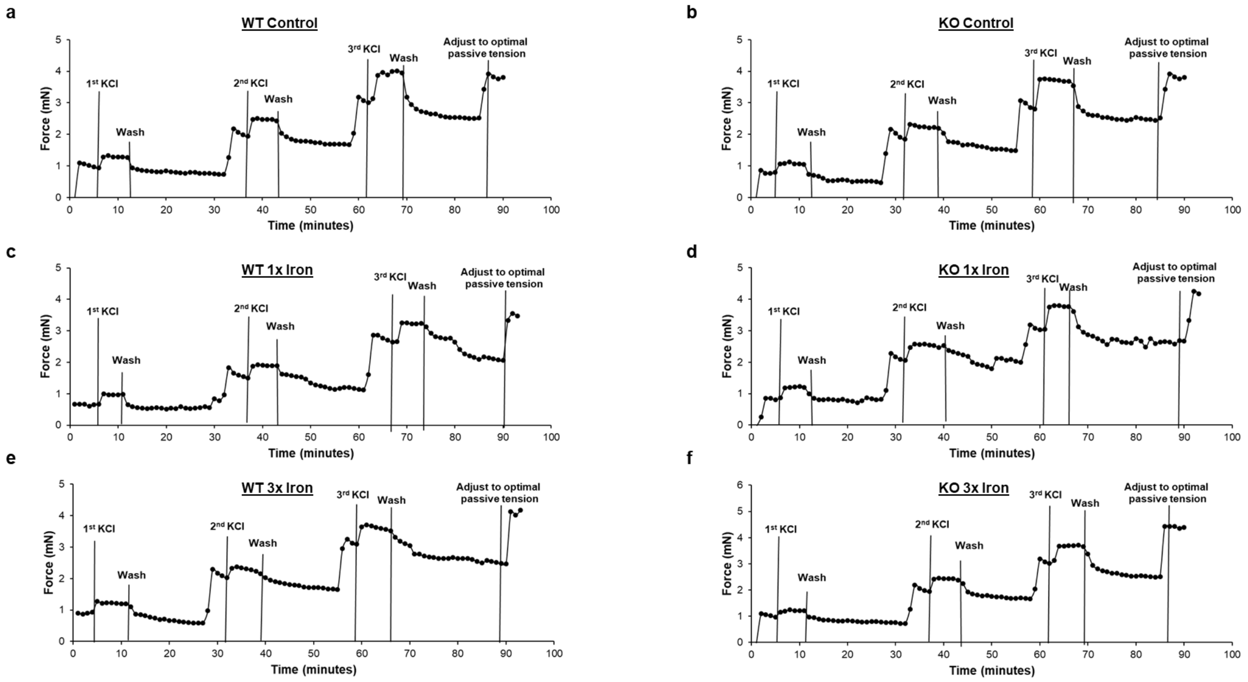

Supplementary Figure S3. Representative original traces of vascular reactivity of WT and CSE-KO aortic rings. Time–force traces show DMT normalization for determining optimal passive tension. (a, b) Representative traces from WT and KO control groups, respectively. (c, d) Representative traces from WT and KO 1× iron groups, respectively. (e, f) Representative traces from WT and KO 3× iron groups, respectively.

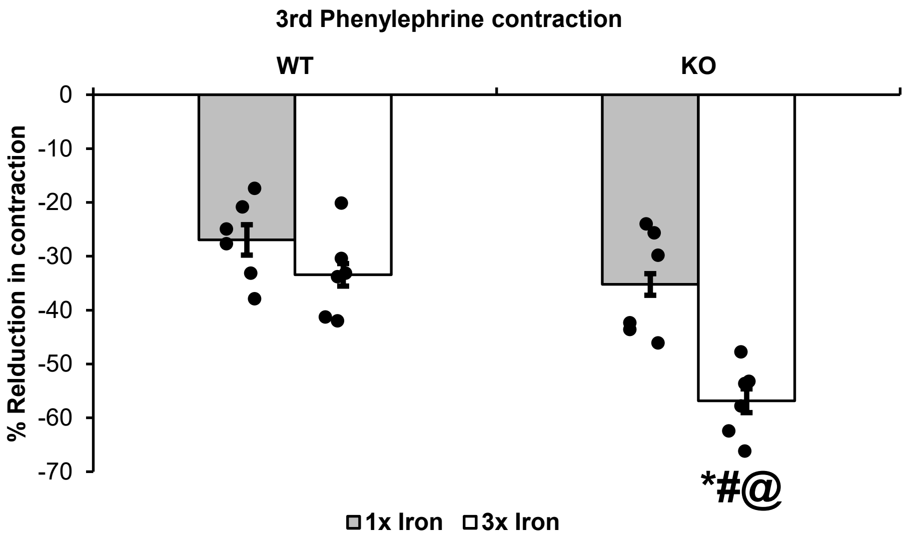

Supplementary Figure S4. Reduction in third phenylephrine (PE)-induced contraction following iron loading in WT and CSE-KO mice. (a) Percent reduction in contraction in WT and KO aortic rings with 1× and 3× iron loading, calculated relative to the respective genotype controls. Bars represent mean ± SEM with individual data points overlaid (n = 6 per group). Effects of mouse genotypes, iron loading concentrations, and their interaction were assessed by two-way ANOVA (Fgenotype(1,20) = 17.86, p = 0.00041; Fconcentrationn(1,20) = 22.70, p = 0.00012; Finteraction(1,20) = 5.17, p = 0.034). Significance symbols represent Tukey's post-hoc comparisons. * p < 0.05 vs. WT 1× iron; @ p<0.05 vs WT 3× iron; # p < 0.05 vs. KO 1× iron.
